# Curcumin ameliorates ischemic stroke injury by downregulating GMFB expression: An in vitro study

**DOI:** 10.17305/bb.2024.10957

**Published:** 2024-09-26

**Authors:** Xiumei Bai, Yabin Song, Xiangyan Zhang, Liqiong Liu, Haixia Wu, Jiaqing Feng, Lihong Wu, Huizhen Liu, Diangui Zhou

**Affiliations:** 1 Pharmaceutical Department, Zhongshan Torch Development Zone People’s Hospital, Zhongshan, China; 2 Department of Neurology, Xiangan Hospital Affiliated to Xiamen University, Xiamen, Fujian, China; 3 Department of Neurosurgery, Shijiazhuang People’s Hospital, Shijiazhuang, China; 4 Department of Oncology, Daqing Oilfield General Hospital, Daqing City, Daqing, China; 5 Department of Circulatory Medicine, Zhongshan Torch Development Zone People’s Hospital, Zhongshan, China; 6 Department of Endocrinology, Zhongshan Torch Development Zone People’s Hospital, Zhongshan, China; 7 Department of Neurosurgery, Zhongshan Torch Development Zone People’s Hospital, Zhongshan, China

**Keywords:** Ischemic stroke (IS), curcumin (Cur), glia maturation factor beta (GMFB), brain microvascular endothelial cells (BMECs), oxygen–glucose deprivation/reoxygenation (OGD/R)

## Abstract

Ischemic stroke (IS) is a cerebrovascular sickness, and cerebral ischemia–reperfusion (I/R) damage often occurs, but there is still a lack of drugs that can significantly alleviate it. Curcumin (Cur) exerts pharmacological effects, such as antioxidative stress, anti-inflammation, and the promotion of apoptosis through regulating various pathways, but its efficacy and specific mechanism of action in IS have not been fully clarified. The purpose of this paper is to study the influence of Cur on IS. Brain microvascular endothelial cells (BMECs) were used to create an oxygen–glucose deprivation/reoxygenation (OGD/R) model to simulate I/R damage. The cell viability was assessed using an MTT assay. The LDH level and ROS positive rate were measured using commercial kits. The cell invasion was examined using a transwell assay. The apoptosis was assessed by flow cytometry. The contents of glia maturation factor beta (GMFB), Bax, and Bcl2 were measured using western blot. We confirmed that in the OGD/R-induced IS cell model, the abundance of GMFB was enhanced in the OGD/R group vs the control group. GMFB overexpression promoted OGD/R-induced cell viability diminution, increased LDH and ROS levels, lessened cell invasion ability, enhanced cell apoptosis, enhanced Bax levels, and decreased Bcl2 levels. Silencing GMFB ameliorated OGD/R-induced cell damage. Cur ameliorated OGD/R-induced cell damage. Cur curbed OGD/R-induced cell damage by downregulating GMFB expression. In conclusion, Cur cured IS-induced cell damage by downregulating GMFB expression.

## Introduction

Stroke has a high incidence and mortality and is the main cause of permanent disability in adults worldwide. It also imposes a heavy economic burden on families and society [1]. Stroke includes haemorrhagic stroke and ischemic stroke (IS), with IS being more common clinically. IS is a cerebrovascular condition caused by insufficient cerebral blood supply, manifested as unilateral limb paralysis, loss of consciousness, and even fainting [2]. Restoring blood supply in the shortest possible time is the primary means to improve the nervous system condition and prognosis of IS patients. However, in most cases, after timely drug or surgical thrombolytic perfusion therapy, the original nerve injury does not recover, and necrosis and excessive apoptosis may occur. This is referred to as cerebral ischemia–reperfusion (I/R) damage [3]. The pathogenesis of cerebral I/R damage is complex, and its clinical manifestations are severe, posing a significant threat to life and creating a heavy economic burden for patients. Despite this, there is still a lack of drugs that can significantly alleviate cerebral I/R injury.

Curcumin (Cur) is a traditional Chinese medicine component extracted from the rhizome of the *Curcuma* genus. As a representative drug of “medicine and food homology,” Cur has a long history of clinical and daily application in China and is often used to treat conditions, such as blood stagnation and amenorrhea, shoulder and back pain, qi and blood disorders, wind-cold, and dampness [4]. Cur exerts pharmacological effects, such as antioxidative stress [5], anti-inflammation [6], antifibrosis [7], and the promotion of autophagy and apoptosis [8] by regulating various pathways, showing great potential and application prospects in clinical disease prevention and treatment. Cur has shown good potential and prospects in the treatment of cerebrovascular conditions, such as IS, haemorrhagic stroke, senile dementia, and the prevention of restenosis after cerebrovascular interventional angioplasty [9]. However, the specific mechanisms of Cur have not been fully clarified, which limits its practical use in clinical practice. Therefore, further basic and clinical research is needed to confirm the specific mechanisms of Cur’s pharmacological action, promote the development of foundational research into clinical applications, and enable Cur to play its full role in the prevention and treatment of cerebrovascular diseases.

Glia maturation factor beta (GMFB) is highly conserved among species and is one of the main members of the GMF family [10]. GMFB is found in mice, rats, zebrafish, and humans and is distributed in the brain, thymus, and small intestine of vertebrates [10]. Although GMFB is highly expressed in the brain, few studies have examined its function, particularly its role in nerve injury and regeneration. Current studies on GMFB focus on degenerative brain diseases and cytoskeletal remodeling. It has been found that GMFB is mainly expressed in mammalian astrocytes and some neurons, promoting the growth and differentiation of nerve cells [10]. Yuan et al. [11] found that GMFB could serve as a reliable marker of ischemic damage in stroke patients. Plasma GMFB may be an indicator of stroke diagnosis and prognosis [11]. However, the effect of GMFB on IS requires further study.

In this paper, brain microvascular endothelial cells (BMECs) were used to establish an oxygen–glucose deprivation/reoxygenation (OGD/R)-induced IS cell model. We constructed overexpression and knockdown vectors of GMFB to study their effects on OGD/R-induced IS cell models. In addition, we further confirmed the influence of Cur on OGD/R-induced IS cells and its regulatory role on GMFB. This study is expected to provide new potential biological therapeutic targets and clinical therapeutic drugs for IS.

## Materials and methods

### BMECs culture

The BMECs were isolated from neonatal Sprague-Dawley rats (within 1–3 days of age; China Center for Type Culture Collection, Wuhan, China). The Sprague-Dawley rats were taken and sterilized after being euthanized (via intraperitoneal injection of pentobarbital sodium at a dose of 100 mg/kg). They were soaked in 75% ethanol (Solarbio, Beijing, China) for disinfection. About 5 min later, the rats were taken out and placed on sterile dry filter paper. The brains were removed by decapitation, and the diencephalon, cerebellum, and white matter of the brain were carefully separated using anatomical forceps. The remaining cerebral hemispheres were rolled back and forth on dry filter paper to remove the blood vessels on the brain’s surface. Finally, the isolated brain tissue was placed in a Petri dish containing pre-cooled D-hank’s solution (Solarbio), and carefully rinsed three times. Then, 2 mL of complete culture solution containing Endothelial Cell Growth Supplement (10% FBS, DMEM/F12; Solarbio) was added to the dish. The removed brain tissue was cut up and transferred to a centrifuge tube for static stratification. After about 3 min, the supernatant was carefully discarded, and 0.1% type II collagenase containing 30 U/mL DNase I (Solarbio) was added to the centrifuge tube. The solution was mixed and digested repeatedly, then centrifuged (4 ^∘^C, 14,000 rpm, 5 min) for about 10 min, and the supernatant was discarded. Next, 2 mL of 20% BSA (Solarbio) was added to the centrifuge tube, and the mixture was blown and mixed repeatedly, then centrifuged (4 ^∘^C, 1000 rpm, 20 min). The upper liquid in the centrifuge tube was discarded, and 2 mL of 0.1% collagenase/dispersozyme (Solarbio) containing 20 U/mL DNase was added into it, and it was blown and digested repeatedly. After about 10 min, the supernatant was centrifuged (4 ^∘^C, 14,000 rpm, 5 min), discarded, and the complete culture medium was added. The mixture was blown and mixed; the inoculation was placed in the culture bottle, and the inoculation status was observed under an inverted microscope (Leica, Wetzlar, Germany). All animal tests were performed according to the *Guide for the Care and Use of Laboratory Animals* [12]. The animal research complied with the Animal Ethical and Welfare Committee of Zhongshan Torch Development Zone People’s Hospital (Approval No. HJRY-2020-01-001).

### Establishment of OGD/R model

BMECs were taken and plated in 96-well plates at a density of 2 × 10^5^/mL and 100 µL per well. The cells were washed to ensure the same background before cell damage in each group. The same amount of sugar-free DMEM medium (Solarbio) was replaced and cultured in an anoxic chamber (5% CO_2_, 95% N_2_) in a conventional incubator for 4 h. Then, the sugar-free DMEM medium was substituted with 10% FBS, DMEM/F12 complete culture medium (Solarbio), and cultured with 5% CO_2_ at 37 ^∘^C for 24 h. The control group cells were cultivated in a complete medium in a conventional incubator, and the supernatant was absorbed and replaced with the fresh medium when the cells were reoxygenated and resugared. The two groups of cells were named control group and OGD/R group, respectively. For Cur treatment, 20 µM Cur (Solarbio, dissolved in DMSO) was added 2 h before OGD/R modeling.

### Cell transfection

GMFB cDNA was cloned into the eukaryotic expression vector pcDNA3.0 (GMFB) and transfected into BMEC cells to regulate GMFB expression. As a control, transfection was performed using pcDNA3.0 without any cDNA insertion (pcDNA3.0). To inhibit the expression of GMFB in BMEC cells, siRNA against GMFB (si-GMFB) and the corresponding control (si-NC) were synthesized. All sequences were synthesized by GenePharma (Shanghai, China). Related plasmids were transfected into BMECs with Lipofectamine 2000 (Invitrogen, Carlsbad, CA, USA). After transfection in a 5% CO_2_ incubator at 37 ^∘^C for 6 h, the cells were washed and recovered in DMEM medium with 10% FBS. Follow-up treatment was performed 48 h after transfection.

### MTT assay

The BMECs were plated in 96-well plates at a density of 2 × 10^5^/mL and 100 µL per well. After OGD/R management, MTT (5 mg/mL; 20 µL/well; Solarbio) was added to each well. After incubating for 4 h, the supernatant was discarded, and DMSO (150 µL/well, Solarbio) was added. The absorbance value at 490 nm was detected using a microplate reader.

### LDH activity and ROS positive detection

After the different treatments, the BMECs were planted in 96-well plates. Following OGD/R management, the supernatant of each well was collected, and the LDH release and ROS positive rates of the cells were detected using the LDH detection kit (Solarbio) and the ROS detection kit (Solarbio).

### Transwell assay

After the different treatments, the BMECs were taken, and transwell assays were performed with an 8 µm pore size polycarbonate membrane (BD Biosciences, Bedford, MA, USA). Briefly, 4 × 10^5^ BMEC cells were resuspended in 100 µL serum-free DMEM in the upper transwell chamber pre-coated with matrix gel. Then, 500 µL of DMEM with 10% of FBS (Solarbio) were added to the lower chamber of the transwell. After 24 h, the cells on the lower surface of the membrane were treated with methanol (Solarbio) for 15 min and stained with crystal violet (Solarbio) for 20 min. Finally, the cell count was determined using an optical microscope (Leica).

### Flow cytometry assay

BMECs were plated in 96-well plates (2 × 10^5^/mL). After culturing for 48 h, the cells were collected, washed, resuspended in PBS, and detected in accordance with the ANNEXIN V FITC/PI Apoptosis Detection Kit (Solarbio) instructions. FlowJo software was used to analyze the data.

### Western blot

Cells were lysed with RIPA lysis buffer (Solarbio), and the protein content was estimated using a bicinchoninic acid assay (Solarbio). Proteins from BMECs were separated and divided by adopting SDS-PAGE. Following this, the samples were moved to a PVDF membrane (Invitrogen). After blocking, the membrane was exposed to antibodies against GMFB (ab303512; 1:1000; Abcam, Cambridge, MA, USA), Bax (ab32503; 1:1000; Abcam), Bcl2 (ab203516; 1:1000; Abcam), and GAPDH (ab8245; 1:1000; Abcam) at 4 ^∘^C overnight. The membrane was then treated with goat anti-rabbit IgG (ab205718; 1:2500; Abcam) for 1 h. Proteins were visualized using an ECL-Plus reagent (Beyotime, Shanghai, China), and the images were analyzed with ImageJ software.

### Ethical statement

The animal research complied with the Animal Ethical and Welfare Committee of Zhongshan Torch Development Zone People’s Hospital (Approval No. HJRY-2020-01-001).

### Statistical analysis

Statistical analysis was performed using GraphPad Prism 9.0. Each experiment was conducted a minimum of six times, and the data followed a Gaussian distribution and were recorded as the mean ± SD showed as Histogram. An unpaired *t*-test was used to examine differences between two groups, and the ordinary one-way ANOVA with post-hoc test was applied to make comparisons between multiple groups. Prism software (GraphPad 9.0) was used for plotting. *P < 0.05, **P < 0.01, ***P < 0.001 indicated that the results were statistically significant.

## Results

### OGD/R-induced IS cell model

**Figure 1.**
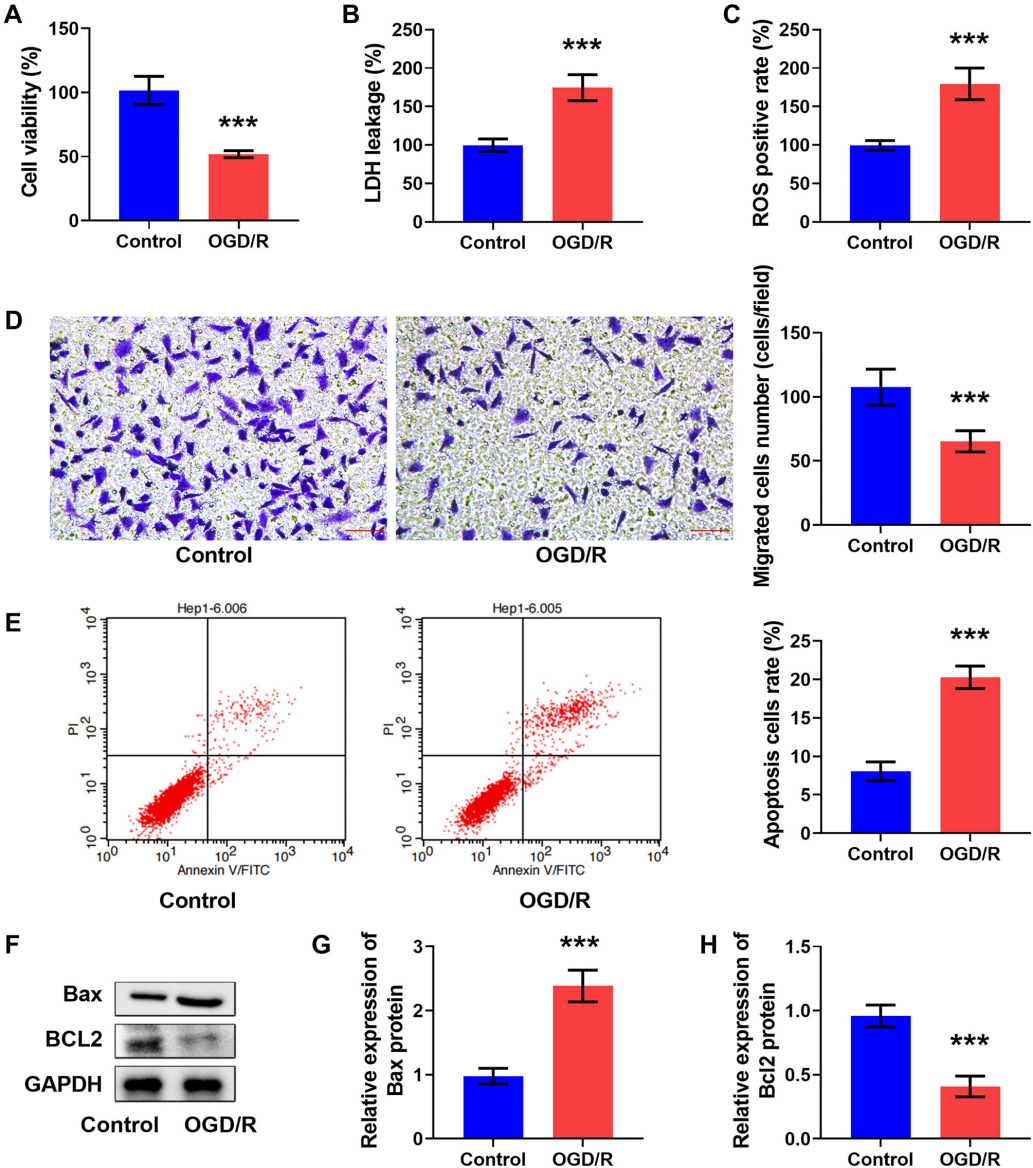
**OGD/R-induced IS cell model.** (A) Cell viability was assessed using the MTT assay. LDH levels (B) and the ROS positive rate (C) were measured using commercial kits. (D) Cell invasion was examined using a transwell assay; scale bar: 40×, 50 µm. (E) Apoptosis was detected by flow cytometry. (F–H) Bax and Bcl2 levels were measured using western blot. ***P < 0.001. The tests were conducted six times (*n* ═ 6). An unpaired *t*-test was used for comparing the differences between the two groups. OGD/R: Oxygen–glucose deprivation/reoxygenation; IS: Ischemic stroke.

BMECs were used to create the OGD/R cell model to simulate cerebral I/R damage. We found that cell viability (see Figure 1A) and cell invasion (see Figure 1D) were reduced, but LDH levels (see Figure 1B) and ROS positive rates (see Figure 1C) were enhanced in the OGD/R group compared to the control group. Additionally, cell apoptosis (see Figure 1E) and Bax levels (see Figure 1F and 1G) were increased, but Bcl2 content (see Figure 1F and 1H) was decreased in the OGD/R group vs the control group. These results confirmed that the OGD/R model was effectively constructed.

### GMFB overexpression promoted OGD/R-induced cell damage

**Figure 2.**
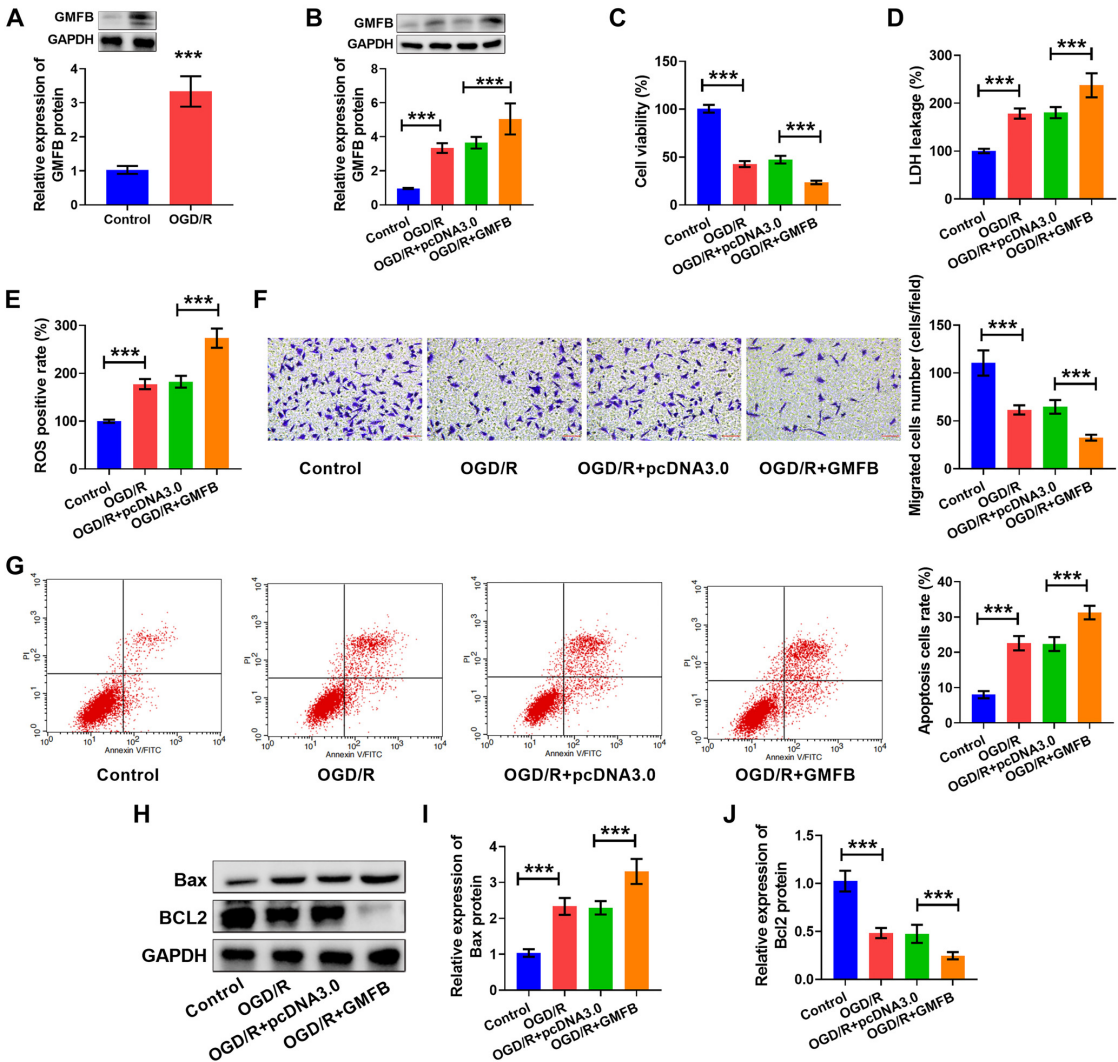
**GMFB overexpression promoted OGD/R-induced cell damage.** (A and B) GMFB content was measured using western blot. (C) Cell viability was assessed using the MTT assay. LDH levels (D) and the ROS positive rate (E) were measured using commercial kits. (F) Cell invasion was examined using a transwell assay; scale bar: 40×, 50 µm. (G) Apoptosis was detected by flow cytometry. (H–J) Bax and Bcl2 levels were measured using western blot. *P < 0.05, **P < 0.01, ***P < 0.001. The tests were conducted six times (*n* ═ 6). An unpaired *t*-test was used for comparing the difference between two groups, and ordinary one-way ANOVA with a post-hoc test was applied to make comparisons between multiple groups. GMFB: Glia maturation factor beta; OGD/R: Oxygen–glucose deprivation/reoxygenation.

In this part, we examined the role of GMFB in OGD/R-induced cell damage. We found that the abundance of GMFB was elevated in the OGD/R group vs the control group (see Figure 2A). This result indicated that GMFB may play a regulatory role in OGD/R-induced cell damage. Further, the abundance of GMFB in OGD/R-induced cells was increased by GMFB transfection (see Figure 2B). Moreover, cell viability (see Figure 2C), cell invasion (see Figure 2F), and Bcl2 content (see Figure 2H and 2J) were reduced, but LDH levels (see Figure 2D), ROS positive rate (see Figure 2E), cell apoptosis (see Figure 2G), and Bax levels (see Figure 2H and 2I) were increased in the OGD/R + GMFB group vs the OGD/R + pcDNA3.0 group. Therefore, we demonstrated that GMFB overexpression aggravated OGD/R-induced cell damage.

### Silencing GMFB ameliorated OGD/R-induced cell damage

**Figure 3.**
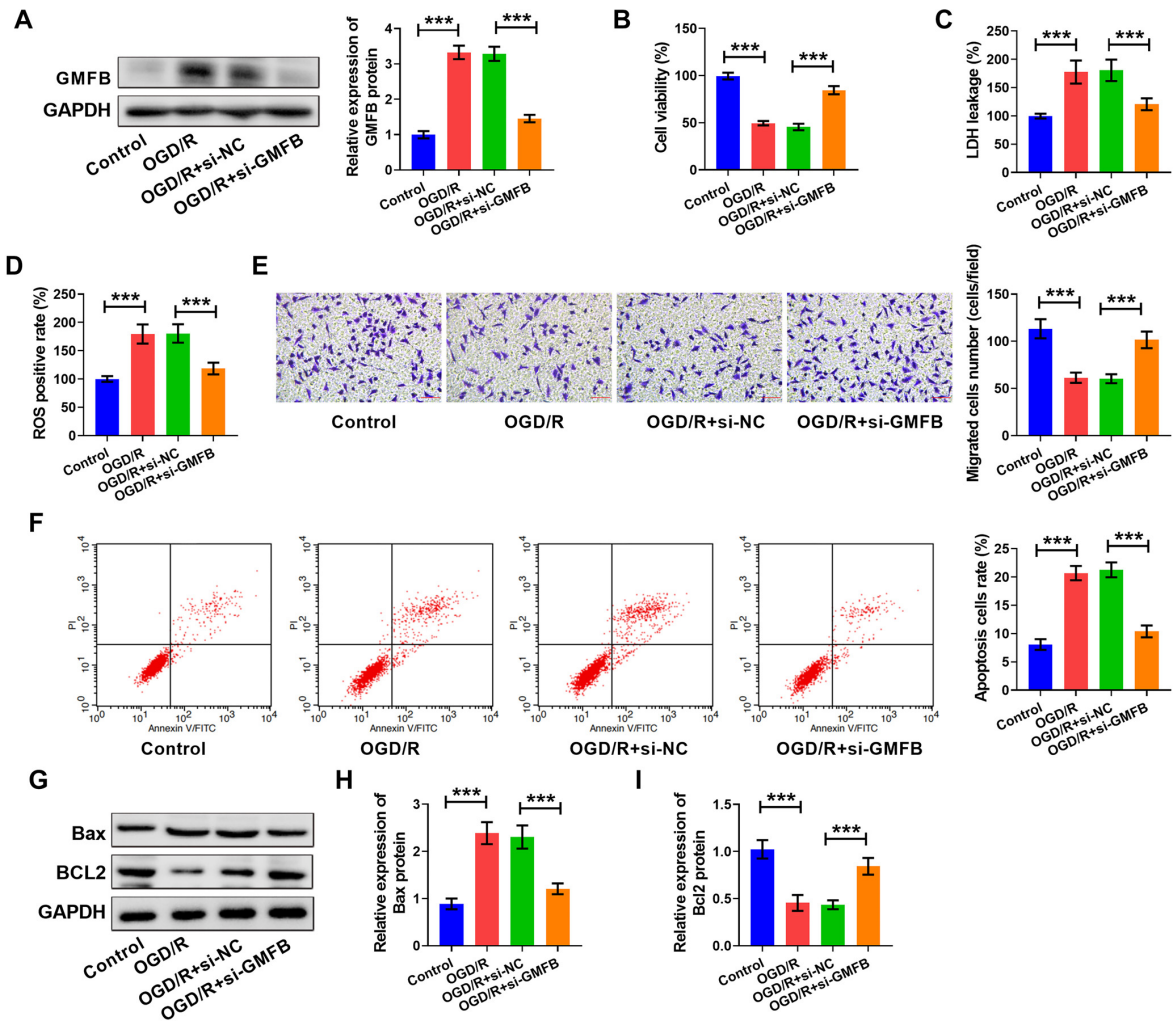
**Silencing GMFB ameliorated OGD/R-induced cell damage.** (A) GMFB content was measured using western blot. (B) Cell viability was assessed using an MTT assay. The LDH levels (C) and the ROS positive rate (D) were measured using commercial kits. (E) Cell invasion was examined using a transwell assay, scale bar: 40×, 50 µm. (F) Apoptosis was detected by flow cytometry. (G–I) Bax and Bcl2 levels were measured using western blot. *P < 0.05, **P < 0.01, ***P < 0.001. The tests were conducted six times (*n* ═ 6). Ordinary one-way ANOVA with a post-hoc test was applied for comparisons between multiple groups. GMFB: Glia maturation factor beta; OGD/R: Oxygen–glucose deprivation/reoxygenation.

We found that the abundance of GMFB in OGD/R-tempted cells was reduced by si-GMFB transfection (see Figure 3A). Additionally, we confirmed that the cell viability (see Figure 3B), cell invasion (see Figure 3E), and Bcl2 content (see Figure 3G and 3I) were enhanced, while LDH levels (see Figure 3C), ROS positive rate (see Figure 3D), cell apoptosis (see Figure 3F), and Bax levels (see Figure 3G and 3H) were reduced in the OGD/R + si-GMFB group vs the OGD/R + si-NC group. Thus, we uncovered that reduced GMFB curbed OGD/R-induced cell damage.

### Cur ameliorated OGD/R-induced cell damage

**Figure 4.**
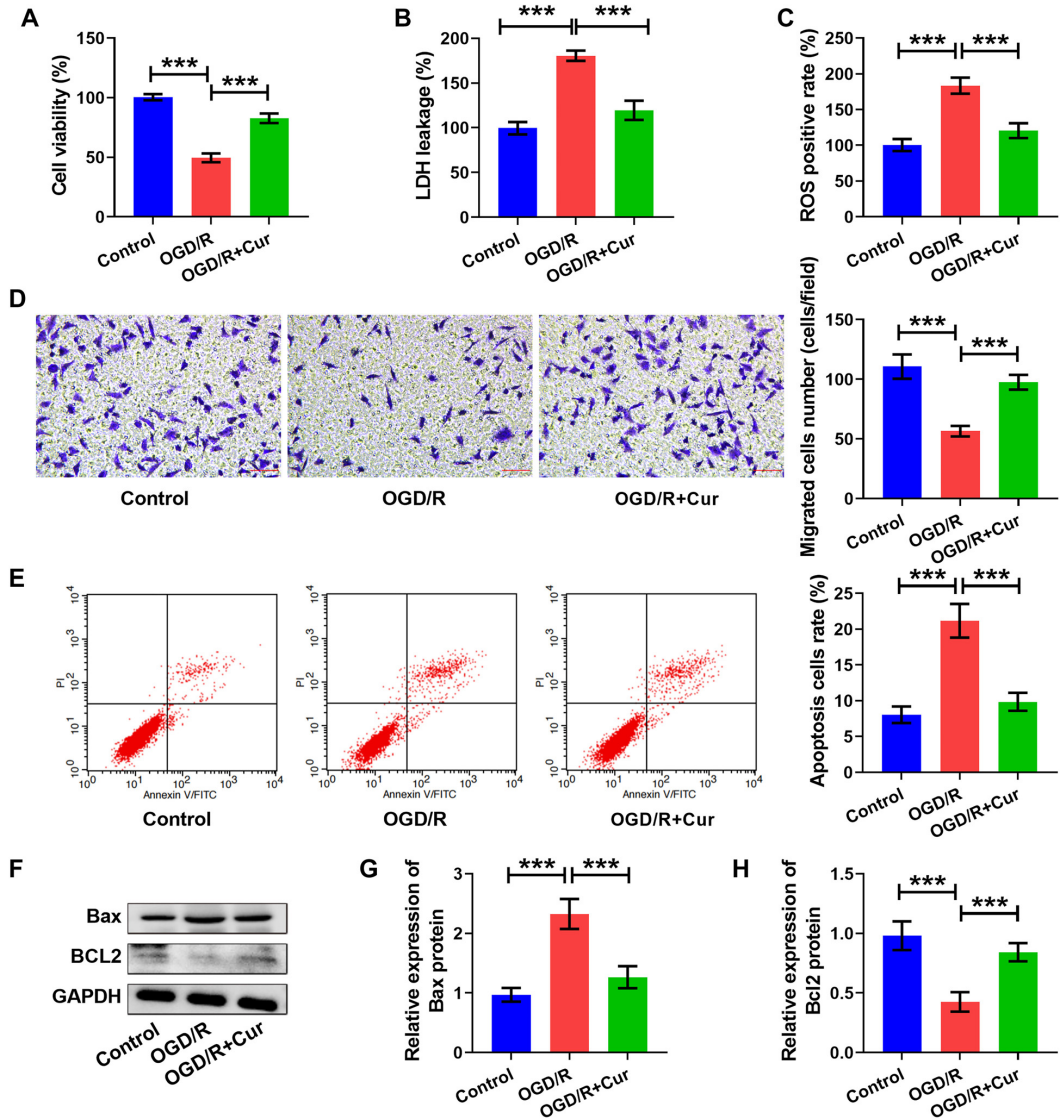
**Curcumin ameliorated OGD/R-induced cell damage.** (A) Cell viability was assessed using the MTT assay. The LDH levels (B) and the ROS positive rate (C) were measured using commercial kits. (D) Cell invasion was examined using a transwell assay; scale bar: 40×, 50 µm. (E) Apoptosis was detected by flow cytometry. (F–H) Bax and Bcl2 levels were measured using western blot. **P < 0.01, ***P < 0.001. The tests were conducted six times (*n* ═ 6). Ordinary one-way ANOVA with a post-hoc test was applied to make comparisons between multiple groups. OGD/R: Oxygen–glucose deprivation/reoxygenation.

We examined the effect of Cur on OGD/R-induced cell damage. We uncovered that cell viability (see Figure 4A) and cell invasion (see Figure 4D) were increased, but the LDH levels (see Figure 4B) and ROS positive rates (see Figure 4C) were decreased in the OGD/R + Cur group vs the OGD/R group. Moreover, the cell apoptosis (see Figure 4E) and Bax levels (see Figure 4F and 4G) decreased, but Bcl2 content (see Figure 4F and 4H) was amplified in the OGD/R + Cur group compared to the OGD/R group. These results revealed that Cur could ameliorate OGD/R-induced cell damage.

### Curc curbed OGD/R-induced cell damage by repressing GMFB

**Figure 5.**
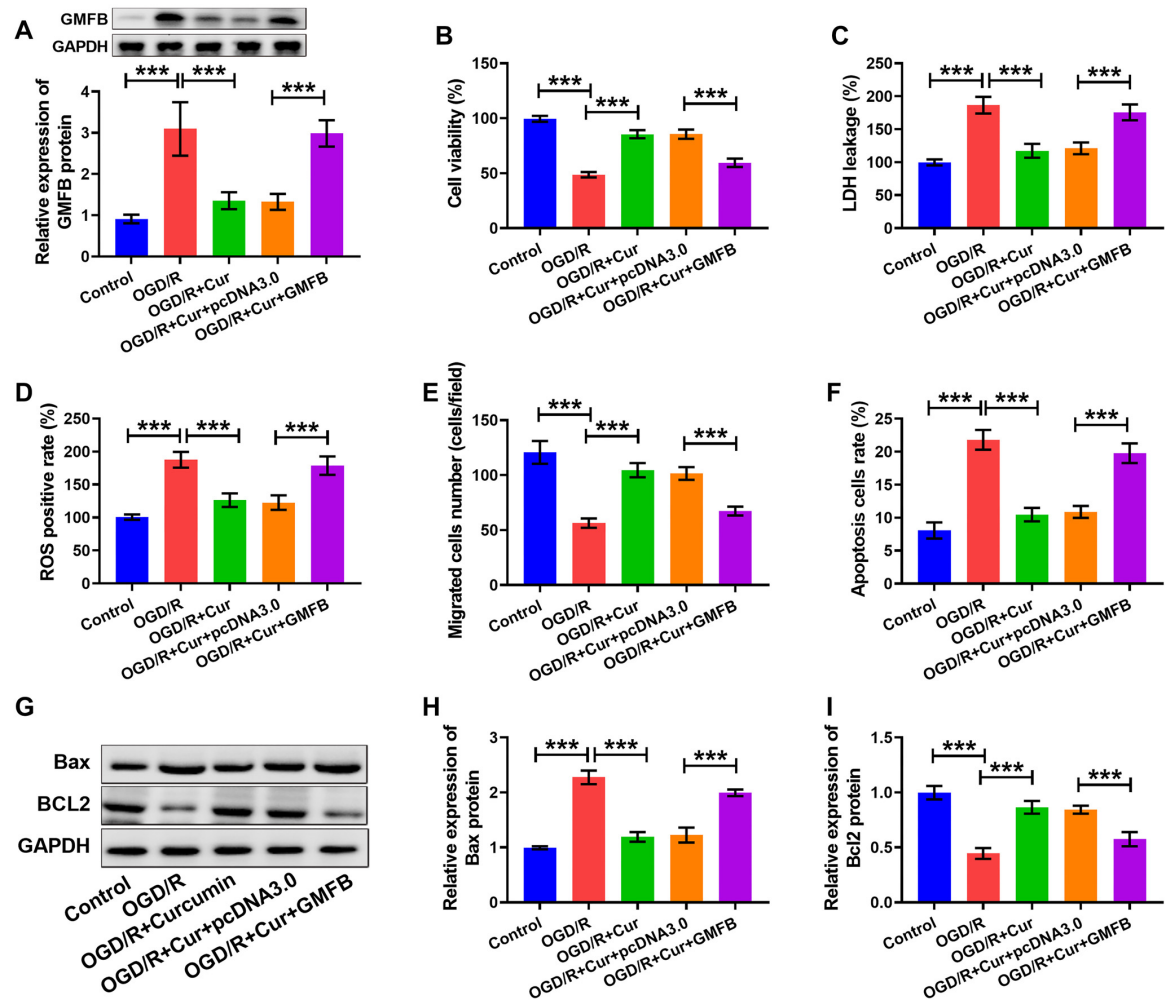
**Curcumin ameliorated OGD/R-induced cell damage by inhibiting GMFB.** (A) GMFB content was measured using western blot. (B) Cell viability was assessed using the MTT assay. LDH levels (C) and the ROS positive rate (D) were measured using commercial kits. (E) Cell invasion was examined using a transwell assay. (F) Apoptosis was detected by flow cytometry. (G–I) Bax and Bcl2 levels were measured using western blot. *P < 0.05, **P < 0.01, ***P < 0.001. The tests were conducted six times (*n* ═ 6). Ordinary one-way ANOVA with a post-hoc test was applied to make comparisons between multiple groups. GMFB: Glia maturation factor beta; OGD/R: Oxygen–glucose deprivation/reoxygenation.

**Figure 6.**
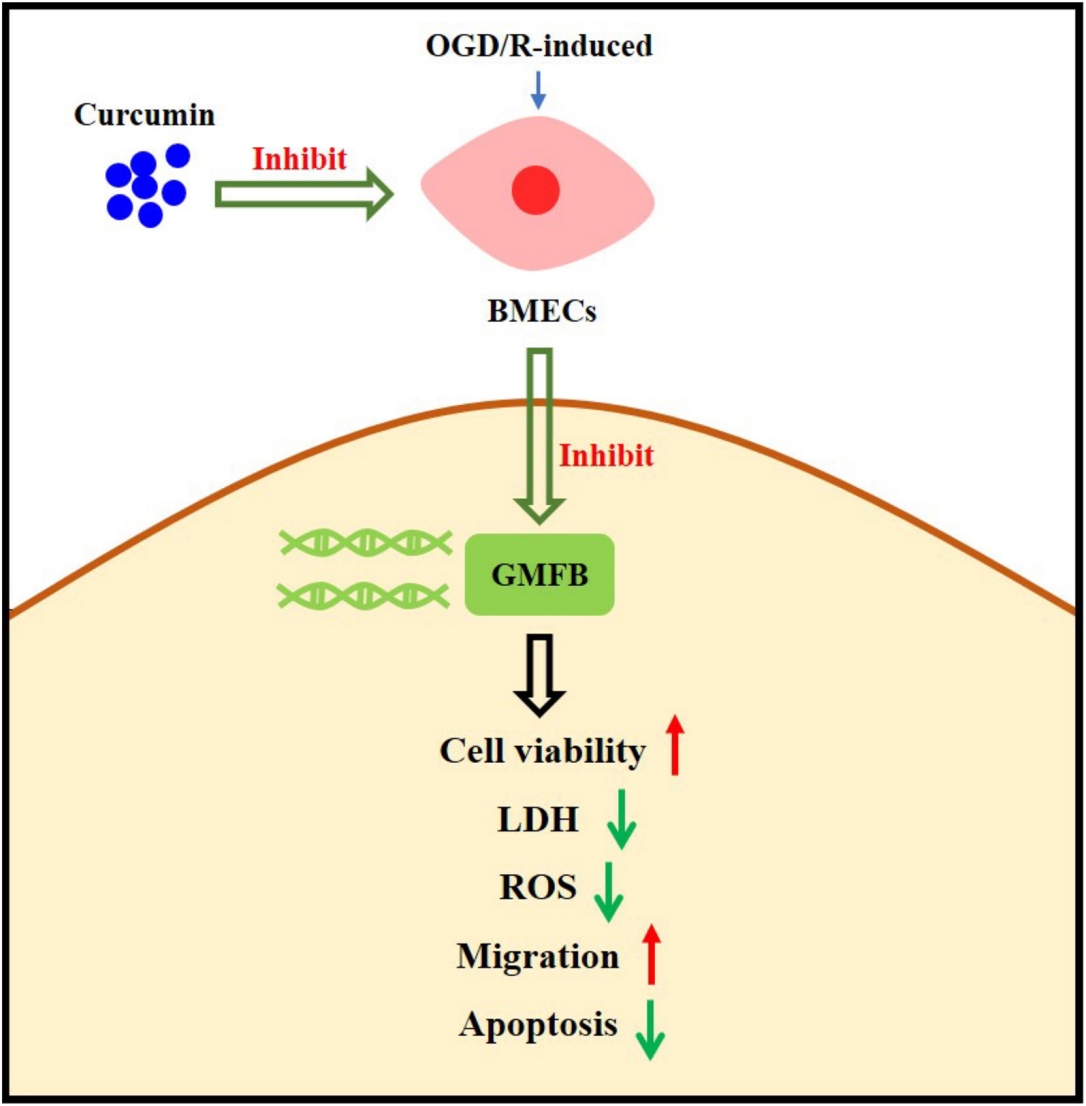
**Schematic diagram of the regulatory mechanism of curcumin in IS.** IS: Ischemic stroke.

In this part, we performed restoration tests. We found that GMFB levels were reduced after Cur treatment, although this effect was diminished by GMFB overexpression (see Figure 5A). Moreover, the cell viability (see Figure 5B), cell invasion (see Figure 5E), and Bcl2 content (see Figures 5G and 5I) were increased, but the LDH levels (see Figure 5C), ROS positive rates (see Figure 5D), cell apoptosis (see Figure 5F), and Bax levels (see Figure 5G and 5H) were reduced after Cur treatment; however, these effects were diminished by GMFB upregulation. The specific regulatory mechanism is shown in Figure 6. These results revealed that Cur curbed OGD/R-induced cell damage by downregulating GMFB.

## Discussion

In this research, we confirmed that the OGD/R-induced IS cell model was successfully established. We revealed that the abundance of GMFB was elevated in the OGD/R group vs the control group. GMFB overexpression diminished OGD/R-induced cell viability, increased LDH and ROS levels, lessened cell invasion ability, increased cell apoptosis and Bax levels, and decreased Bcl2 levels. Silencing GMFB ameliorated OGD/R-induced cell damage, and Cur was shown to curb OGD/R-induced cell damage by inhibiting GMFB expression. In conclusion, Cur curbed OGD/R-induced cell damage by downregulating GMFB expression.

Cerebral I/R injury, induced by IS, refers to brain cell damage caused by mitochondrial dysfunction, oxidative stress, inflammatory infiltration, glutamate toxicity, Ca^2+^ overload, programmed cell death, and other mechanisms, resulting in a large decrease in the number of nerve cells and significant impairment of brain function [3]. The metabolic requirements of brain tissue for oxygen and glucose are much higher than those of other tissues and are almost exclusively dependent on oxidative phosphorylation. Reduced blood flow in the brain disrupts the transport of substances (especially oxygen and glucose) and the production of energy necessary to maintain the ion gradient [13]. This leads to decreased production of high-energy phosphorylated substances; increased production of free radicals and mitochondrial swelling, ridge breakage, and cytochrome C release occurred, leading to apoptosis [14]. In the center of focal cerebral ischemia, cerebral blood flow is reduced by only 20%, and hypoxic depolarization develops rapidly, leading to cell death due to fat and protein decomposition, microtubule depolymerization after bioenergy depletion, and disruption of ion balance in vivo [15]. Current clinical work requires new therapeutic strategies.

GMFB is an acidic protein extracted from brain tissue, mostly expressed in astrocytes and some neurons in the central nervous system. Xu et al. [16] found that overexpression of GMFB in astrocytes could increase ROS activity and lead to oxidative stress, while the antioxidant stress ability of astrocytes in GMFB-knockout mice was increased, and the release of inflammatory factors was reduced. Bcl2 inhibits cell apoptosis, and animal experiments showed that apoptosis occurred in acute myocardial infarction, accompanied by decreased expression of Bcl2 [17]. In this research, we found that GMFB was upregulated in the OGD/R group vs the control group. This result indicated that GMFB may play a regulatory role in OGD/R-induced cell damage, similar to the findings of Yuan et al. [11]. Moreover, we revealed that GMFB overexpression diminished OGD/R-induced cell viability, LDH and increased ROS levels, lessened cell invasion ability, enhanced cell apoptosis, increased Bax levels, and decreased Bcl2 level. Silencing GMFB curbed OGD/R-induced cell damage. These findings were comparable to the result of Xu et al. [16].

Wu et al. [18] studied the effect and mechanism of Cur in treating IS in a rat model, and the effects revealed that Cur can significantly upregulate the abundances of ZO-1 in cerebrovascular endothelial cells and Occludin and Claudin-5 in the blood–brain barrier. The development of blood–brain barrier function can significantly reduce the ischemic infarct area of the brain, promote the synaptic remodeling of hippocampal neurons, reduce neural function deficits, and improve the nervous system score of rats. Ran et al. [19] found that in a rat model of spinal cord injury, Cur reduces the content of proinflammatory factors and reactive gliosis in the rat brain stem by inhibiting the inflammatory response of microglia, preventing the formation or expansion of glial scar areas. In addition, the study found that Cur ameliorates white matter injury after IS by inhibiting microglia/macrophage pyroptosis [19]. Salt-inducible kinase 3 in microglia/macrophages can regulate normal and excessive phagocytosis after transient focal cerebral ischemia [20]. These studies indicate that Cur plays a vital role in the occurrence, progression, treatment, and prognosis of IS and is an important drug for the management of IS. The mechanism of its action may be related to the improvement of vascular endothelial function. In this research, we found that Cur ameliorated OGD/R-induced cell damage, consistent with the findings of Wu et al. [18]. In addition, we were the first to discover that Cur curbed OGD/R-induced cell damage by suppressing GMFB expression. However, there are still some limitations in this research. We only studied the regulatory role of Cur in animal cell models. In a follow-up study, we will develop an animal model and further verify the therapeutic effect of Cur on IS in clinical settings.

## Conclusion

In conclusion, our findings revealed that Cur ameliorated OGD/R-induced cell damage by downregulating GMFB. Therefore, this study has strong clinical translational potential and application prospects. It will expand our understanding of the pathogenesis of IS and further strengthen the theoretical foundation for targeted IS treatment.

## Supplemental data

**Highlights**

- GMFB overexpression promoted OGD/R-induced cell injury.
- Silencing GMFB curbed OGD/R-tempted cell injury.
- Curcumin curbed OGD/R-tempted cell damage by downregulating GMFB.

## Data Availability

The data supporting the findings of this study can be obtained from the corresponding author, upon request.
